# Decoding Melanoma Development and Progression: Identification of Therapeutic Vulnerabilities

**DOI:** 10.3389/fonc.2020.626129

**Published:** 2021-02-04

**Authors:** Kevinn Eddy, Raj Shah, Suzie Chen

**Affiliations:** ^1^Graduate Program in Cellular and Molecular Pharmacology, School of Graduate Studies, Rutgers University, Piscataway, NJ, United States; ^2^Susan Lehman Cullman Laboratory for Cancer Research, Rutgers University, Piscataway, NJ, United States; ^3^Joint Graduate Program in Toxicology, Rutgers University, Piscataway, NJ, United States; ^4^Rutgers Cancer Institute of New Jersey, New Brunswick, NJ, United States; ^5^Environmental & Occupational Health Sciences Institute, Rutgers University, Piscataway, NJ, United States

**Keywords:** melanoma, melanoma progression, metastasis, signaling pathways, melanoma therapies

## Abstract

Melanoma, a cancer of the skin, arises from transformed melanocytes. Melanoma has the highest mutational burden of any cancer partially attributed to UV induced DNA damage. Localized melanoma is “curable” by surgical resection and is followed by radiation therapy to eliminate any remaining cancer cells. Targeted therapies against components of the MAPK signaling cascade and immunotherapies which block immune checkpoints have shown remarkable clinical responses, however with the onset of resistance in most patients, and, disease relapse, these patients eventually become refractory to treatments. Although great advances have been made in our understanding of the metastatic process in cancers including melanoma, therapy failure suggests that much remains to be learned and understood about the multi-step process of tumor metastasis. In this review we provide an overview of melanocytic transformation into malignant melanoma and key molecular events that occur during this evolution. A better understanding of the complex processes entailing cancer cell dissemination will improve the mechanistic driven design of therapies that target specific steps involved in cancer metastasis to improve clinical response rates and overall survival in all cancer patients.

## Introduction to Melanoma

In the United States, cancer is the second leading cause of death and is expected to surpass heart disease in a few years (1). Skin cancer is by far the most common of all cancers, with an increasing frequency in the past three decades that includes basal cell carcinoma (BCC), squamous cell carcinoma (SCC), and melanoma. Although melanoma accounts for merely 1% of all skin cancers, it is responsible for the majority of skin cancer related fatalities. Melanoma is the most aggressive and dangerous forms of skin cancer that develops from the transformed pigment forming cells of the skin, melanocytes (2). Diagnosing melanoma in its early stages, *in situ*, is crucial for the prognosis and survival of this deadly disease as the 5-year survival rate for primary melanoma is 99% and for metastatic melanoma is only 27% (1). Global incidence rates for melanoma have steadily increased over the years; in the United States approximately 100,000 new cases of invasive melanoma will be diagnosed in 2021 and about 7,000 of those melanoma patients will die from this disease (1, 3). There are various risk factors associated with melanoma: a family history of skin cancer, being a male, fair skin, number of moles, age, and UV exposure (1, 3–9). The most common inherited genetic defects associated with a predisposition to developing melanoma are the cell cycle regulating genes: *CDKN2A*, *CDK4*, a gene responsible for skin pigmentation: *MC1R*, and the genetic disorder xeroderma pigmentosum (XP) that disrupts the proper repair of UV induced DNA damage thereby leading to a higher mutation rate (10–17).

A dermatologist usually diagnoses melanoma on a patient using the ABCDEF criteria with the help of a dermascope, a tool that removes skin surface reflections to accurately distinguish between a benign or malignant lesion (18–20). The ABCDEF criteria are: Asymmetry, Border irregularity, Color variegation, Diameter >6 mm, Evolution of a nevi and Funny looking, where a malignant nevi does not conform to the common profiles of nevi found on a patient (18). Once diagnosed, the melanoma is staged using a set of principles developed by the American Joint Committee on Cancer (AJCC) to guide patient treatment and prognosis (21). Melanoma patients can be classified into five distinct stages, 0, I, II, III, and IV, as the stage increases the prognosis is worse (21). Stage 0 is defined as melanoma *in situ* while stage IV melanoma is known as metastatic melanoma. Metastatic melanoma is defined by the dissemination of primary melanoma cells to distant organs including but not limited to the lymph nodes, lungs, liver, brain, and bone (21, 22). AJCC criteria uses different permutations of the TNM system, to categorize melanoma from early stage to late stage melanoma (21). The TNM system is defined as: Tumor thickness with or without ulceration, Nodal involvement, and Metastasis (21). Great advances have been made in the understanding of melanoma pathogenesis that have resulted in improved disease treatment outcomes including targeted therapies: BRAF and MEK inhibitors and immunotherapies: monoclonal antibodies that target CTLA-4, PD-1 and PD-L1, however not all patients respond, and resistance eventually develops to these agents. This underscores the importance of dissecting the molecular pathways mediating metastasis, the processes of transitioning of an immobile melanoma cell into a motile cell that can successfully colonize distant organs. A better understanding of these pathways will help in the identification of biological markers (biomarkers) for better diagnosis and provide rational therapeutic strategies to predict favorable treatment responses.

## Tumor Intrinsic and Extrinsic Functions Necessary for Successful Tumor Cell Dissemination

### Stepwise Molecular Evolution for the Transition of Primary Melanoma to Metastatic Melanoma

**Figure 1:**
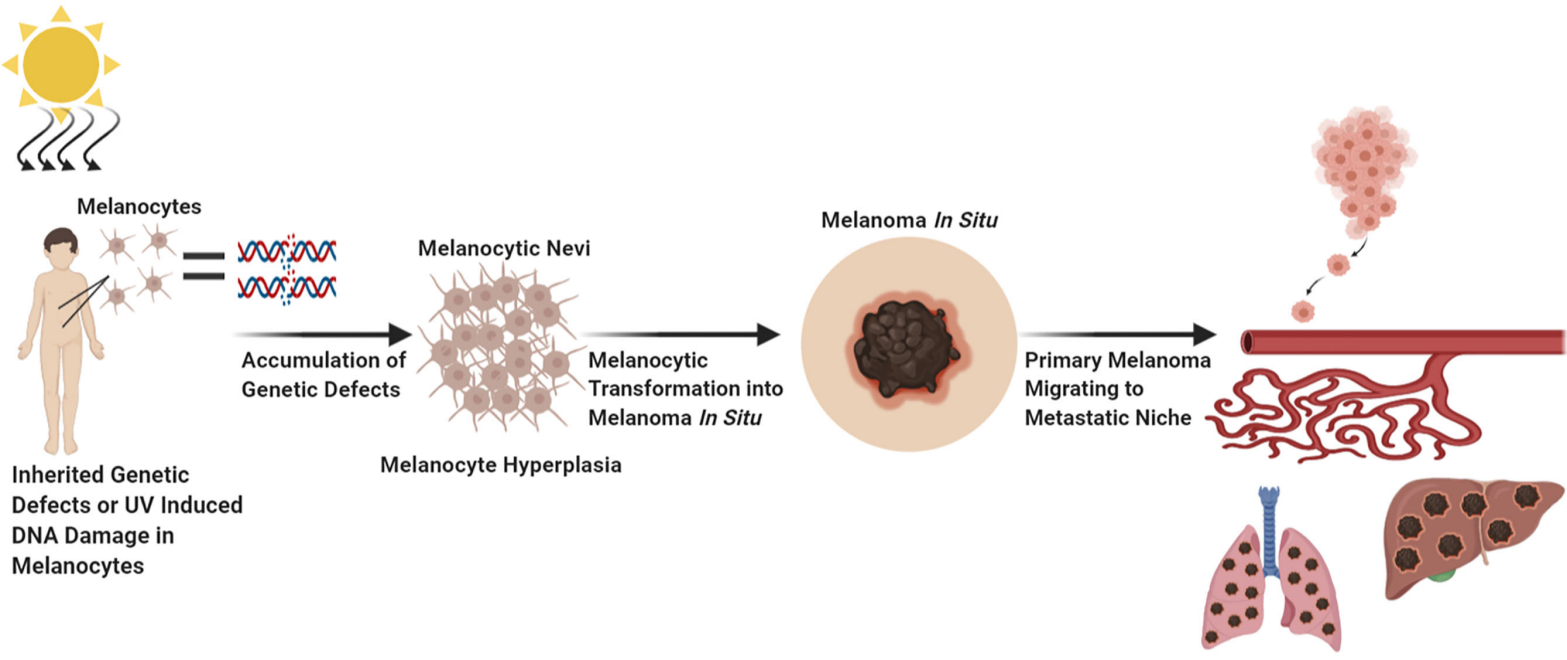
Factors Which Contribute to Melanocytic Transformation. Created with BioRender.com.

Melanoma has the highest mutational burden of any cancer as a result of UV induced DNA damage and/or DNA replication errors (8, 23). All these mutations contribute to various aspects of melanocytic neoplasia; however, certain mutations are considered driver mutations as they are likely to initiate melanocytic transformation, the early steps of tumor formation, progression, and dissemination. Vogelstein et al., and Shain et al., have elegantly described the genetic evolution transpiring during the transformation of a melanocytic lesion into malignant melanoma (**Figure 1**) (6, 24, 25). First a normal melanocyte acquires an initiating driver mutation that leads to melanocyte hyperplasia and nevi development (6, 25–28). These steps are known as the breakthrough phase, with a low mutational burden, and copy number alterations (24, 25). Common mutations found in melanocyte nevi are *BRAF* mutations (26–28). Mutations in *BRAF* and *NRAS* are frequently mutually exclusive, with *NRAS* mutations sometimes found in nevi, especially congenital nevi (29, 30). The next step known as the expansion phase where some of the melanocytic nevi progress into intermediate lesions and overtime develop into melanoma *in situ*, that is accompanied with the acquisition of the *TERT* promoter mutations, and a high mutational burden (6, 24, 25, 31). The *TERT* gene encodes for telomerase reverse transcriptase, the catalytic component of telomerase, an enzyme required for the maintenance of telomeres (31). Aberrant expression of telomerase allows melanoma cells to become replicative immortal (31). After the accumulation of various mutations such as: *CDKN2A*, *TP53*, *PTEN*, and genes encoding SWI/SWF chromatin remodeling complex subunits, primary melanoma enters the invasive phase and becomes malignant melanoma (6, 24, 25). This phase is characterized by a high tumor mutational burden and increased copy number alterations (6, 25). To note, only 20–40% of melanomas arise from nevi and the rest are *de novo*, however *de novo* melanomas may arise from clinically undetectable precursor lesions, and these lesions may follow similar trajectory as detectable lesions (**Figure 1**) (6, 25, 32, 33).

In addition to the genetic defects associated with metastatic melanoma development there are several dysregulated key signaling pathways that occur during melanoma progression such as the WNT, MAPK, and PI3K/AKT pathways (22, 34, 35). These pathways are involved in melanoma cell proliferation, growth, survival, evading cell death, and acquiring metastatic properties (34, 35). The MAPK and PI3K/AKT pathways can cooperate with each other in the transduction of survival signals (36). The WNT signaling cascade plays a fundamental role in embryogenesis (37). The involvement of such a central pathway, the WNT pathway, in melanoma cell dissemination suggests that the reactivation of elements associated with embryogenesis is crucial in elucidating cancer cell metastases (37, 38). Embryogenesis requires a single cell to proliferate and differentiate into various cell types and acquire migratory/invasive properties required for body patterning that parallels carcinogenesis (37, 38). Signal transduction of the WNT, MAPK, and PI3K/AKT pathways in melanoma cells promotes altered expression of cell adhesion molecules and peptidases allowing for the remodeling of the extracellular matrix (ECM) to facilitate in cancer cell migration (34, 39–42). During melanoma progression, elevated matrix metallopeptidase (MMP) expression and function have been detected (34, 43, 44). MMP facilitates the degradation of the ECM that supports melanoma growth during early stages and eventual migration to distant organs (34, 43, 44). Increased MMP avidity in the tumor microenvironment is contributed by both tumor production of MMP as well as tumor induced fibroblast production of MMPs (39–47). Loss of the ECM enables melanoma cells to become anchorage independent and anoikis resistance that support melanoma dissemination through the circulatory system. In addition to MMP cleaving connections between melanoma cells and ECM, the loss of adhesion molecules such as integrins and cadherins also contribute to the motility of melanoma cells from the primary site (22, 34, 48–51). Cell adhesion molecules are required for cell attachment to the basement membrane and cell-cell interactions allowing for the proper development of tissues and organs. Under normal physiology, cell adhesion molecules, integrins and E-cadherins are involved in the attachment of melanocytes to the basement membrane and mediating the interactions between keratinocytes and melanocytes (52, 53). During melanoma progression, E-cadherins are progressively reduced to allow for the dissociation between melanocytes and keratinocytes followed by concomitant upregulation of N-cadherins to support melanoma cell survival, and migration through tissues, a process regulated by the PI3K/AKT pathway (54–56). In addition to modulations in the expression of cadherins during metastasis, integrins can be modulated to support motility and migration into hospitable metastatic niches by modifying basement membrane interactions, supporting angiogenesis formation and expression of MMPs (34, 51, 57–59). There are specific micro RNAs (miRNA), metastamiRs, that were shown to potentiate cancer cell metastasis by regulating critical steps associated with epithelial and mesenchymal transition (EMT), angiogenesis, colonization, adhesion, migration, and invasion (60). Melanoma cell interactions with neighboring cells are essential for their survival, proliferation, and dissemination, in line with this we will discuss the importance of immune evasion in melanoma metastasis.

### Interactions Between the Host Immune System and Melanoma Cells to Support Melanoma Cell Growth and Dissemination

**Figure 2:**
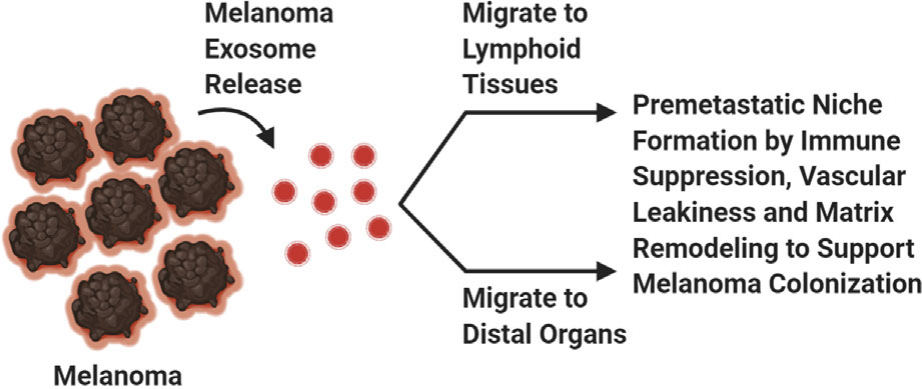
Exosomes. Melanoma exosomes create a pre-metastatic niche at distal sites to support melanoma cell dissemination. Created with BioRender.com.

Our immune system is essential for defending us from foreign pathogens that invade our body. Cancer is a distorted version of our normal self, and under this guise it can evade immune destruction through the process of immune editing (61, 62). It takes many years through the process of immune editing for a clinically detectable melanoma (or other cancers) to emerge (61, 62). Immune editing is composed of three phases: elimination, equilibrium, and escape phases (61, 62). Immunogenic melanoma clones during the elimination phase are detected by antigen presenting cells, phagocytosed and cross-presented to melanoma specific cytotoxic T-cells for activation to induce an anti-tumor responses against melanoma associated antigens (61). This process inherently allows for the selection of low immunogenic melanoma cell clones to survive and evade host immune detection while highly immunogenic cell clones are eliminated, a process termed as the equilibrium phase (61). Immune resistant melanoma cell clones that have survived are able to proliferate and migrate to distant organs without immune detection, a term coined as the escape phase (61). The process of immune editing is supported by the notion that the first site of melanoma metastasis is detected in the lymph nodes, an organ composed of many cytotoxic immune cells (**Figure 2**) (22). If the immune system is not suppressed, then these melanoma cells would not be able to survive and thrive at this site (**Figure 2**). The migration of low immunogenic primary melanoma cells into lymph nodes may not be attributed solely to the passive migration of these cells from the primary niche to the metastatic niche, but rather a preferential migration to the lymph nodes to improve tumor fitness and colonization abilities (63–65). The migration of melanoma cells from the skin to the lymph nodes is attributed to their ability to secrete soluble factors, in addition to the presence of small vesicles, exosomes, which contain cargo that promote the lymphatic system to expand its vasculature (65) (**Figure 2**). Furthermore, lymphatic endothelial cells secrete cytokines and chemokines that support the movement of tumor cells to the lymph nodes (65). All these factors are key contributors to the successful colonization of melanoma cells to the lymphatic tissues (65). One of the rate limiting steps in the establishment of distant metastasis is oxidative stress (65). To overcome this barrier, primary melanoma cells preferentially migrate to the lymph nodes where they are educated to become resistant to oxidative stress (65). This adaptation allows these tumor cells to successfully seed and colonize distant organs compared to circulating melanoma cells (64, 65). Next, we will discuss the critical players in tumor metastasis including genetic mutations, signaling pathways, tumor microenvironment and the involvement of small vesicles, exosomes (**Figure 2**).

### Experimental Models to Study Melanoma Metastasis

Experimental models that recapitulate the onset and progression of human disorders is essential in biomedical research to bridge the gap between basic science and the treatment of diseases. There are very few animal models for tumor metastasis, with the B16 mouse melanoma cell line being a very popular one. The B16 parental cell line was derived from a C57BL/6 mouse which spontaneously developed lesions and were subsequently adapted to grow in cultured conditions (66). There are several subclones of B16 cells with differing metastatic capabilities (66, 67). These subclones were derived by subcutaneously injecting B16 cells into syngeneic C57BL/6 mice (spontaneous metastasis), or by intravenous injection of these cells into circulation (experimental metastasis) (66). Our spontaneous melanoma-prone mouse model is driven by the ectopic expression of a normal neuronal receptor, Metabotropic Glutamate Receptor 1 (protein: mGluR1; gene: *GRM1*) in melanocytes which is a useful model to study spontaneous metastasis in a biologically and physiologically relevant manner (68–72). We demonstrated this melanoma-prone mouse model has several advantages: it develops spontaneous metastatic melanoma with 100% penetrance, the progression of the disease mimics human melanoma progression and metastatic dissemination with melanomas being detectable first in the lymph nodes and at later stages in the lung, brain, and other sites (68–72). In some cases, the melanotic tumors can undergo phenotypic changes into amelanotic metastatic tumors similar to human melanomas (72). Recently, Kos and co-workers used fluorescence imaging to trace melanoma cell dissemination in an intact *in vivo* setting using crosses of our mice, this new strain will be a useful tool to study spontaneous metastasis (73). The study of spontaneous metastasis is hindered since the required interval for spontaneous metastasis to occur *in vivo* takes much longer than the commonly used intravenous inoculation of tumor cells, in addition animal wellness rules in almost all institutions, frequently discourages the practice to keeping tumor-bearing mice for a long period of time.

## Mechanisms and Routes for Melanoma Metastasis

### EMT-to-MET Transition

**Figure 3:**
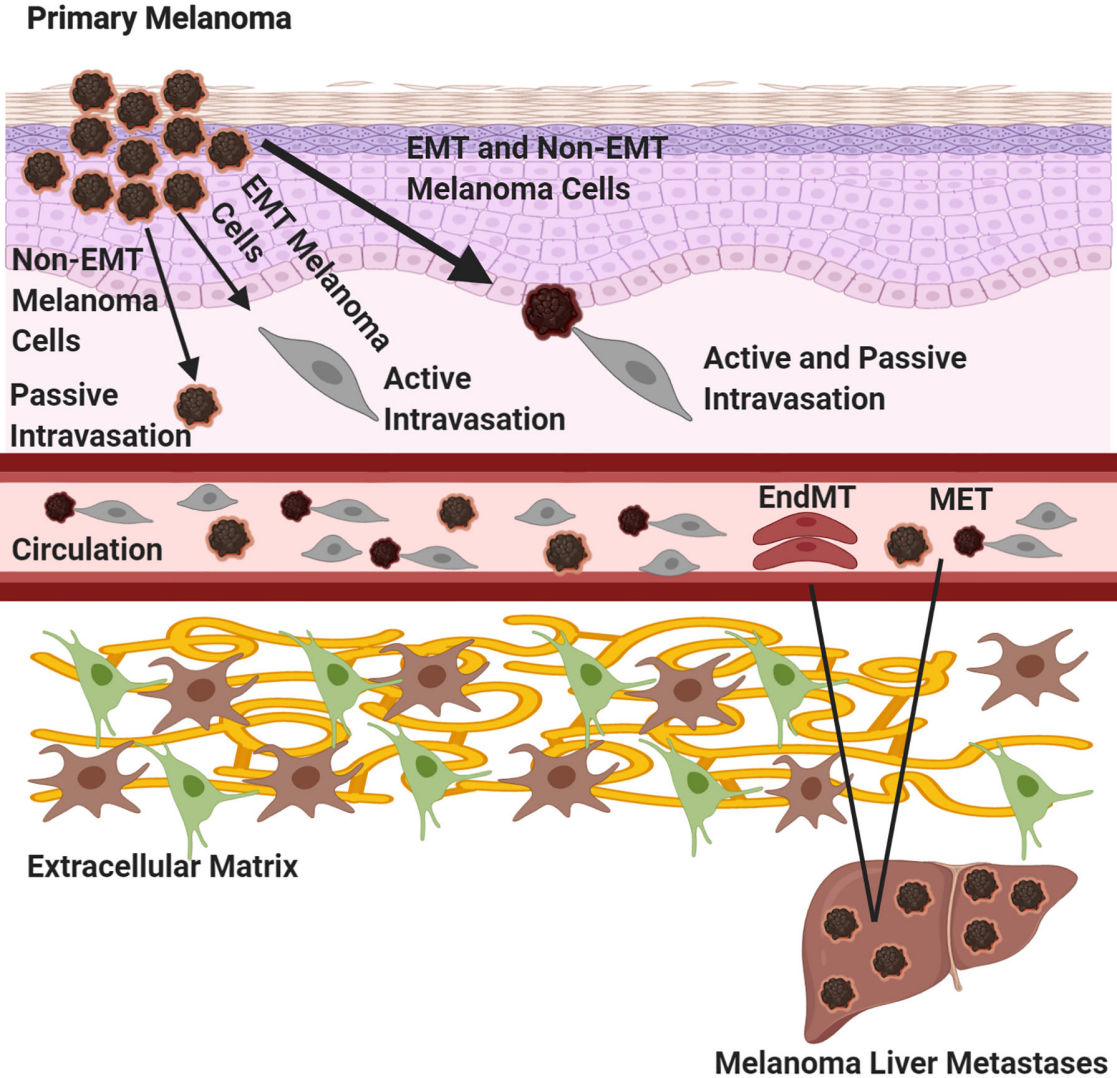
Melanoma Metastasis. Three routes of primary melanoma dissemination are outlined. A primary melanoma can undergo 1) passive shedding of tumor cells, non-EMT (epithelial to mesenchymal transition) followed by passive intravasation, 2) tumor cells can undergo EMT and active intravasation or 3) melanoma cells can undergo EMT and bring along non-EMT tumor cells, where the EMT cells are actively intravasating while the non-EMT cell are undergoing passive intravasation. Once in circulation, tumor cells will migrate to site of metastasis. If the tumor cells are active, they will undergo the canonical extravasation by mesenchymal to epithelial transition (MET). If the tumor cells are dormant, they will transdifferentiate into endothelial cells at the intravascular niche, undergo endothelial to mesenchymal transition (EndMT) and extravasate into the niche. Created with BioRender.com.

There are numerous steps required for melanoma cells, as well as other cancer cells, to successfully spread to distant organs. Melanoma cells must first dissociate from the primary tumor and undergo Epithelial Mesenchymal Transition (EMT), a process by which epithelial cells undergo morphological and phenotypic changes that allow them to become more migratory and invasive through tissues and enter circulation. Tsuji and colleagues have proposed that tumor cells that have not undergone EMT, termed non -EMT tumor cells are attached to EMT tumor cells and “come along for the ride” to distal organs (**Figure 3**) (74). Bockhorn and colleagues suggested the notions of passive and active intravasations (75). In passive intravasation tumor cells are passively shed during tumor progression as a result of a highly stressful environment (**Figure 3**) (75). Active intravasation is when cancer cells are actively undergoing molecular alterations to a metastatic phenotype and follow a chemokine gradient to arrive at the site of metastasis (**Figure 3**) (75). We propose that both passive and active intravasations occur as tumor progresses, since the surrounding extracellular matrix (ECM) is degraded and enables both EMT and non-EMT tumor cells to intravasate into circulation (**Figure 3**) (74, 75). In this scenario, EMT cells are actively intravasating into circulation and non-EMT cells are the passengers as in passive intravasation (**Figure 3**) (74, 75). Once in circulation, these traveling melanoma cells will migrate to their preferential metastatic organs mediated by chemotaxis of specific chemokine ligand-receptor interactions or by passive migration (22, 74, 75). In circulation, melanoma cells can transdifferentiate into endothelial cells where they remain dormant at the intravascular niche near the metastatic site (**Figure 3**) (73). Quiescent in-transit melanoma cells are resistant to therapies, suggesting that these melanoma cells may have transdifferentiated into endothelial-like cells and contribute to melanoma relapse in patients who have previously responded to therapy (73). Interestingly, it was shown that highly metastatic melanoma cells can form their own vascular tubes to improve blood flow to the tumor site and promote cancer cell dissemination, a phenomenon known as vascular mimicry (76, 77). It has been proposed that these transdifferentiated quiescent melanoma cells may undergo an endothelial to mesenchymal transition (EndMT) to extravasate into the metastatic niche (73). Therefore, it is possible that there are at least two distinct mechanisms for circulating melanoma cells to successfully establish at the secondary site depending on if they are active or dormant cancer cells: 1) the canonical extravasation or 2) the proposed transition of a quiescent-like endothelial melanoma cell to convert into a mesenchymal phenotype in order to successfully extravasate into the metastatic niche (**Figure 3**) (73, 78). If the environment of the metastatic niche is favorable, melanoma cells can successfully colonize there and become clinical detectable tumors. Alternate mechanisms of tumor cell dissemination have been proposed. One of these mechanisms is that melanoma metastasis occurs in parallel with the primary tumor rather in a stepwise manner, a distinct idea from EMT (6). This concept is based on the observation that in some melanoma patients with localized melanomas who have had their sentinel lymph nodes removed but did not show improvement in their survival suggests that the tumor cells may already have migrated to distal organs (6, 63, 79, 80). Furthermore, patients who have localized melanomas or no metastasis at all have shown the presence of circulating melanoma cells (6, 81). Another intriguing proposal is that benign melanocyte nevi can migrate to distal sites and acquire oncogenic mutations enabling their transformation into melanoma cells at the metastatic site, a process known as benign metastasis (6, 82–84). Benign metastasis may explain how 4% of melanoma patients develop “metastasized” melanomas despite the absence of detectable primary tumors (6). These findings further complicate our understanding of melanoma metastasis; however, it is possible that in some cases both stepwise and parallel melanoma dissemination occur simultaneously.

### Angiogenesis

**Figure 4:**
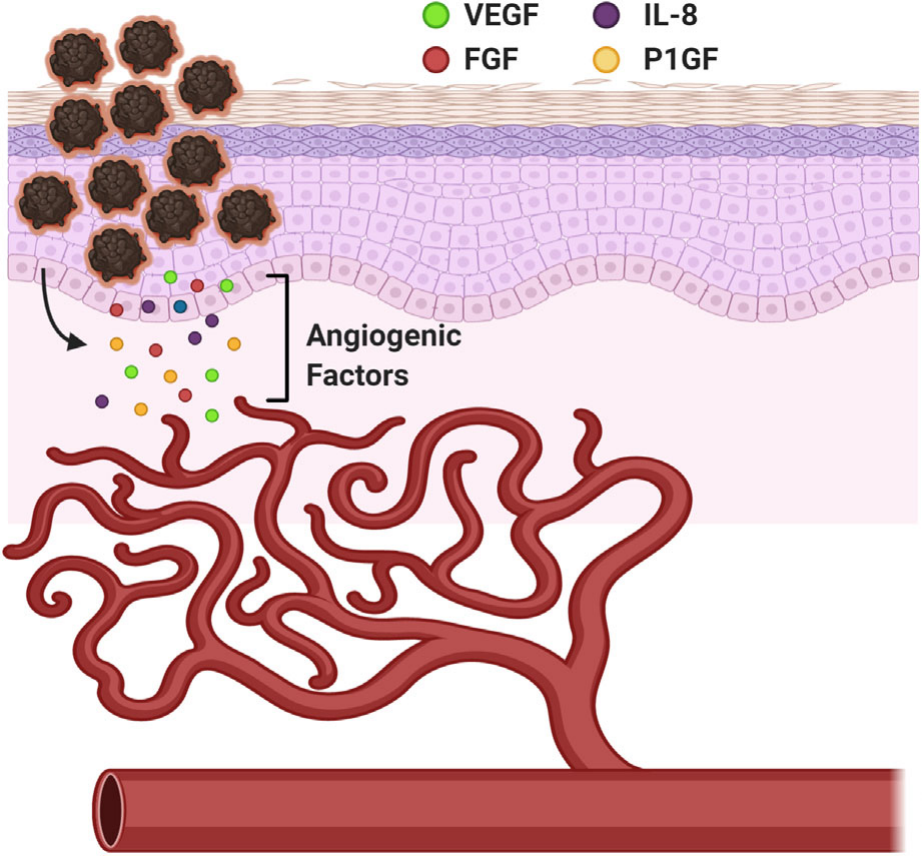
Angiogenesis in Melanoma. A mechanism to provide nourishment to the growing tumor cells and establish routes to distant metastatic sites. Vascular Endothelial Growth Factor (VEGF), Fibroblast Growth Factor (FGF), Interleukin-8 (IL-8), and Placental Growth Factor (PlGF). Created with BioRender.com.

Angiogenesis is a biological process that marks a critical stage in tumor progression where the cells transition from an avascular to a vascular phase, serving as a turning point in melanoma tumor growth and metastasis (**Figure 4**). Melanoma cells serve as the source of several classical angiogenic growth factors including but not limited to vascular endothelial growth factor (VEGF), also known as the vascular permeability factor (VPF), fibroblast growth factor (FGF), interleukin-8 (IL-8), and placental growth factor (PlGF), all potent contributors of angiogenesis (**Figure 4**) (85). VEGF is often considered to be one of the most important mediators of angiogenesis and was shown to have elevated expression in all known solid tumors including malignant melanoma (86). Melanoma cells produce and secrete VEGF into the extracellular matrix (87). Expression of a specific VEGF isoform in an otherwise non-tumorigenic, non-VEGF expressing melanoma cell line results in an aggressive tumor with a highly extensive supporting vasculature, suggesting its undisputed role in promoting angiogenesis (88). Upregulation of IL-8 and VEGF have also been postulated to contribute to the development of resistance to chemotherapeutic agents in melanoma (89). VEGF mediates its effects by interacting with and stimulating its high-affinity transmembrane family of tyrosine kinase receptors, VEGF receptors (VEGFRs). At the molecular level, interactions between VEGF and VEGFR-mediated signal transduction, promote reprogramming of specific gene expression in endothelial cells, including upregulated expression of several proteins encompassing the procoagulant tissue factor, proteins associated with the fibrinolytic pathway, MMPs and a number of anti-apoptotic factors (90, 91). The consequences of this altered gene expression includes stimulation in endothelial cell proliferation and migration, lumen formation, increased vessel dilation and permeability thereby enabling constant supply of both oxygen and nutrients to support the growing tumor (92). Inhibition of angiogenesis through targeting of various driver genes, predominantly VEGF, has been touted as a novel alternative or supplement to conventional cancer therapy (93–95). Since anti-angiogenic agents were shown to effectively slow the growth and metastasis of human melanoma, it is not surprising that the efficacy of these agents in augmenting the benefits of other promising therapies is being tested in clinical trials (87, 96). Unfortunately, anti-angiogenic monotherapies in melanoma did not show remarkable clinical responses and it has been suggested that vascular mimicry plays an important role in improving blood supply to the tumor to support its growth and dissemination (97, 98). It is possible that delivering therapeutics that block angiogenesis or vascular mimicry to early-stage melanoma patients, may impede metastasis in two ways: 1) block nutrient and oxygen flow to the primary tumor and 2) hamper primary tumor cells’ dissemination by inhibiting entry into circulation.

### Exosomes

All cell types release exosomes but cancer cells release higher amounts of exosomes compared to their normal counterparts (2). Cancer exosomes play important roles in creating a favorable environment for cancer cells to thrive in; which can be attributed to the suppression of an anti-tumor immune response and establishment of a pre-metastatic niche (**Figure 2**) (2). Accumulating evidence suggests that the horizontal transfer of tumor exosomal cargo, composed of nucleic acid, lipids, and proteins, into recipient cells within lymphoid tissue promotes immune suppression resulting in defective antigen presentation, reduced antigen specific anti-tumor immune response and upregulation of immunosuppressive cytokines that support melanoma metastasis to the lymph nodes and beyond (**Figure 2**) (99–105). These unique features plus PD-L1 expression on the exosome surface, contribute to defective immune effector cell function at both local and systemic levels (106–108). In addition to their roles in modulating the immune system, other functions have been attributed to exosomal cargo including enhanced vascular leakiness, fibronectin deposition, and delivery of soluble factors that are involved in ECM remodeling to support formation of a metastatic niche for tumor cells, including melanomas (**Figure 2**) (2, 104, 109–112). Exosomes support the “seed and soil” hypothesis of cancer cell dissemination (111–113). Melanoma exosomes located at the most common sites of metastasis, lymph nodes, liver, and lungs, create a “fertile soil” for melanoma cells to “seed” upon arrival then proliferate and manifest into a malignant tumor (111–113). In our mGluR1 driven melanoma model, we have demonstrated that mGluR1^+^ melanoma exosomes when taken up by mGluR1^−^ recipient cells promote these cells to become more migratory, invasive and develop an anchorage-independent growth phenotype compared to mGluR1^−^ melanoma exosomes (114). Taken together, it is clear that every step of tumor dissemination is critical for its successful colonization to distant sites. Better understanding of these necessary molecular and theoretical steps will provide rational therapeutic designs to improve the efficacy of treatments as well as reduce disease relapse.

## Biomarkers in Malignant Melanoma

Melanoma biomarkers can be divided into different categories based on their level of expression compared to normal tissues as well as their ability to serve as prognostic or predictive markers (115). These markers can be further classified into two groups (serum-specific and tissue-specific) depending on their dominant location of expression. Due to the complexity and heterogeneity of melanoma tumors, immunohistochemical staining for tissue-specific melanocytic markers is often used to diagnose melanoma. Microphthalmia-associated transcription factor (MITF), a predominant tissue-specific marker, plays a critical role in lineage commitment of melanocytes and melanoma (116). Normal melanocyte differentiation and proliferation are under the regulation of MITF. MITF expression is also essential for melanoma cell proliferation and survival (117). With integrative genomic analysis, it was found to be amplified in ~16% of melanomas. *BRAF*^V600E^ mutation together with ectopic expression of MITF has been shown to transform primary melanocytes into malignant melanoma (118). In addition, MITF also stimulates the cell cycle regulator, *INK4A*, for efficient melanocyte differentiation (119). Several studies have investigated the potential of MITF in specificity and sensitivity in distinguishing melanoma from other cancers, however, the discovery of the presence of MITF in other non-melanocytic cell types in the tumor microenvironment has complicated this notion (120–122). Similar concerns pertaining to other tissue-specific biomarkers including tyrosinase, MMPs, cyclooxegenase-2 (COX-2), chondroitin sulfate proteoglycan 4 (CSPG4), and human melanoma black-45 (HMB-45) among others also have been reported (123). A lack of “tumor-specific” non-invasive and affordable tools including specific antibodies have greatly hampered the use of biomarkers in diagnosis, prognosis, and prediction of treatment outcomes. It is critical to improve biomarker discoveries and detection tools. Promising candidates are “OMICS” studies that include a variety of cancer and normal tissue specimens along with machine learning approaches may have the potential to promote such findings.

Regarding serum-specific biomarkers, lactate dehydrogenase (LDH) is one of the best prognostic factors in metastatic melanoma (124). Cancer cells including melanoma employ a different metabolic strategy than normal cells to satisfy their energy requirements and sustain cellular proliferation. Under aerobic conditions, normal cells acquire their energy primarily from the conversion of glucose to pyruvate by a process known as glycolysis that occurs in the cytosol. Pyruvate then enters the tricarboxylic acid (TCA) cycle where it converts into carbon dioxide in the mitochondria *via* oxygen-consuming cellular respiration (125, 126). However, under hypoxic conditions such as in most tumors, where oxygen is not readily available, cells prefer to rely more on anaerobic glycolysis that converts glucose into lactate instead of pyruvate. Elevated levels of LDH, an enzyme that catalyzes the conversion of pyruvate to lactate, in systemic circulation was shown to predict survival in patients with metastatic melanoma (127). The increase in serum LDH is associated with poor survival, one of the consequences of melanoma cells outgrowing and surpassing the blood supply (124). Similar to tissue-specific biomarkers, differential sensitivity and specificity are also reported in serum-specific markers including but not limited to LDH, S100 and melanoma-inhibitory activity (MIA) (128). The importance of cancer exosomes in mediating tumor progression has led many investigators to evaluate its diagnostic and prognostic value as a biomarker (2). Cancer exosomes that carry specific molecules such as, PD-L1, CD63, Caveloin-1, MIA, S100B, Glypican-1, and non-coding RNA to name a few, were shown to stratify patients participating in various clinical trials into responders versus non-responders, healthy controls and disease-free patients *versus* cancer patients, and/or cancer patients with differing survival outcomes (104, 107, 129–133). Taken together, the challenges that confront the identification of a reliable melanoma biomarker emphasizes the need to investigate and validate emerging biomarker candidates in the clinic to realize their diagnostic, prognostic, and predictive values.

The identification of a reliable predictive clinical biomarker is crucial for precision medicine. Predictive biomarkers are biological molecules detected in most patients and are frequently correlated with treatment responses (134). Personalized/precision medicine is the future for human disease treatments, and it is essential to identify clinically relevant biomarkers that can be easily applied in the clinic. Most pre-clinical cancer studies only assess for the efficacy of drug(s) in tumor progression, but it is crucial to also identify predictive biomarkers for treatment responses. Identification of these biomarkers will provide clinicians with the opportunity to make suitable and rational decisions in therapeutic options.

## Melanoma Treatments

### Chemotherapies and Targeted Therapies

For patients diagnosed with primary melanoma, surgical removal of the tumor(s) provides the best chance of definitive cure. Late-stage melanoma is difficult to treat due to metastasis, refractile to most treatment modalities and a high genomic variability of a heterogeneous melanocytic tumor (135). The understanding of how various genetic mutations are associated with the onset and progression of melanoma allows for innovation and subsequent implementation of novel therapeutic strategies targeting specific oncogenes. Within the last decade, much progress has been made in the treatment of metastatic melanoma. Earlier studies showed that treatment with Sorafenib (BAY 43-9006), a general multi-kinase inhibitor resulted in inhibition of melanoma cell proliferation *in vitro* and *in vivo* (136). However, in the Phase 2 randomized Sorafenib clinical trial little or no anti-tumor activity was detected in advanced melanoma patients, therefore, the trial was discontinued (137). A highly selective small molecule inhibitor, Vemurafenib/Zelboraf (PLX4720/PLX4032), against cells that harbor the most common mutation in melanoma, mutated *BRAF*^V600E^, was initially reported to have therapeutic effects in patients with advanced melanoma but its effectiveness was marred by patient relapse within 8–12 months (138–141). The treatment responses were short-lived due to the reactivation of the MAPK pathway and/or other mutations (36, 142–144). Combining BRAF inhibitors with other small molecule inhibitors that target other components of the MAPK pathway such as MEK and ERK appear to be an improvement over single-agent therapy but also has increased toxicities (36, 145, 146). It is noteworthy that until recently, it had not been possible to develop an inhibitor towards RAS (147). Christensen et al., reported a KRAS^G12C^ inhibitor that demonstrated pronounced tumor regression in multiple *KRAS*-mutant tumor models (148). *KRAS* mutations are rare in melanoma, where it accounts for about 1.7% and is almost exclusively in codon G12 however it is not known if the recently developed KRAS^G12C^ inhibitor will have any effects in *KRAS* mutated melanomas, furthermore, possible efficacies of the mutated KRAS inhibitor toward *NRAS* mutated melanomas was not tested (149). Despite the nominal successes described above for some patients they only represent a minority of all patients.

### Immunotherapies

Melanoma is one of the most immunogenic types of cancers, hence making it a strong candidate for immune checkpoint blockade (ICB) therapy (150). This therapy utilizes a patient’s own immune system to attack cancer cells with a robust anti-tumor response, long-term immunity, and durable survival. The concept of immunotherapy has been around for approximately 130 years with the early usage of Coley’s toxin, then almost a century later, the uses of interferon (IFN), high dose interleukin- 2 (IL-2) and the cancer vaccine, Bacillus-Calmette-Guerin (BCG) have been described for the treatment of melanoma (151–153). These early immunotherapies were non-specific, however within the past decade the utilization of targeted immunotherapies have risen with monoclonal antibodies that block immune checkpoint molecules, such as cytotoxic T-lymphocyte associated protein–4 (CTLA-4), programmed cell death protein-1 (PD-1), and programmed death ligand-1 (PD-L1), adoptive T-cell therapies and oncolytic viruses (151, 153–155). These new targeted immunotherapies have shown remarkable anti-tumor immune responses with improved survival; however, they only benefit a subset of patients.

During infection, immune activity is heightened in order to properly identify and eliminate the source of infection. To reduce the likelihood of the development of autoimmunity the body uses immune checkpoints such as CTLA-4, PD-1, and PD-L1 to rein in the overactive immune response (156–161). Cancer cells utilize these immune checkpoints to induce local and systemic immune suppression. Since cancer is a chronic disease, T-cells within the lymph nodes are continuously exposed to cancer antigens resulting in the upregulation of CTLA-4 on their cell surface and inhibition of proper T-cell activation, disrupting anti-tumor cytotoxic T-cell functions that results in T-cell anergy (162–164). The PD-1/PD-L1 axis functions within the tumor microenvironment, the PD-1 receptors are expressed on the surface of T-cells and tumor cells express its ligand, PD-L1 (152). The PD-1/PD-L1 axis inhibits cytotoxic T-cell response against tumor cells (152). Interestingly, it was shown that this axis contributes to T-cell anergy within tumor draining lymph nodes and that PD-1/PD-L1 interactions within tumor draining lymph nodes can be used as a prognostic marker to determine melanoma treatment outcomes (165). Monoclonal antibodies were developed to block these immune checkpoint interactions. Ipilimumab blocking CTLA-4, and Pembrolizumab/Nivolumab blocking PD-1. The FDA has approved these antibodies for patients with unresectable or metastatic melanoma and these agents were shown to have strong durable responses with improved survival in a subset of patients (151, 152, 166–169). Stage III melanoma patients who have had their melanomas resected can undergo different regimens based on their *BRAF* genotype (170). Patients who harbor mutated *BRAF* are given adjuvant anti-PD-1 therapy in combination with BRAF and MEK inhibitors, while patients with wild-type *BRAF* are given anti-PD-1, as opposed to anti-CTLA-4 due to toxicity (170). The anti-PD-L1 antibody, Atezolizumab has been approved for unresectable or metastatic melanoma patients with the *BRAF V600* mutation in combination with BRAF and MEK inhibitors, Vemurafenib and Cobimetinib (153). It is not surprising that various combinatorial studies utilizing various permutations of drugs to combine with anti-PD-1/anti-PD-L1 may improve patients’ responsiveness to immune checkpoint blockade therapy have been one of the most sought-after clinical trials in human cancers including melanoma (169).

## Discussion and Future Perspectives

A better understanding of the metastatic processes that govern migration of primary melanoma to distant metastatic niches such as lymph nodes, liver, lung, and brain can aid in the clinical development of novel anti-cancer treatments in the future. Several therapies that target driver pathways of melanoma metastasis have been developed: BRAF and MEK inhibitors, anti-angiogenic therapies and immunotherapies that rejuvenate the immune system to detect and eliminate cancer cells. These therapies have remarkable efficacy in the outcomes of treating malignant melanoma in the past decade, however only a small subset of patients respond. This implies that our understanding of melanoma progression is incomplete. To improve our understanding of the signaling cascades involve in melanoma progression (or other cancers) we suggest conducting a large-scale unbiased biomarker serum profiling screen of healthy donors, disease free patients and melanoma patients at different melanoma stages. Biomarkers are molecules that are linked to disease pathogenesis and identification of biomarkers will reveal biological pathway(s) involved in melanoma progression. Identification of upregulated or downregulated serum markers such as nucleic acids, proteins, exosomes, lipids, and circulating tumor cells may unravel novel or key metastatic pathways that can be further dissected, and therapies developed against them. This proposed concept mirrors forward genetics, we know the external phenotype is melanoma, therefore doing a high throughput un-biased screen for serum biomarkers will reveal expression changes of biomarkers between healthy controls, disease-free patients, and melanoma patients, which will provide insights into key driver pathways regulating metastasis. Using this approach will be the nucleation point to further dissect these pathways and develop a more robust anti-metastatic drug with better responses than current therapies. Furthermore, the identification of a reliable melanoma biomarker that can accurately predict disease treatment outcome in patients and correctly identify patients who will benefit from therapy is still underway. Since melanoma is a molecularly complex and heterogeneous disease with intra- and inter-tumoral variabilities, evaluating multiple biomarkers simultaneously may improve the accuracy and precision of predictive markers than each individual marker.

## Author Contributions

Conceptualization, KE, RS, and SC. Writing—Original Draft Preparation, KE and RS. Writing—Review and Editing, KE, RS, and SC. Supervision, SC. Funding Acquisition, KE, RS, and SC. All authors contributed to the article and approved the submitted version.

## Funding

This work was funded by New Jersey Commission on Cancer Research (NJCCR) Pre-Doctoral Fellowship (DCHS19PPC027) to KE, National Cancer Institute Small Business Innovation Research (NCI SBIR) (R44CA156781-04) to SC, Veterans Administration Research Merit Award (101BX003742) to SC. SC and RS are grateful for the support of the NIEHS T32 training grant in Environmental Toxicology (ES007148). The authors also declare that this study received funding from the Bristol-Myers Squibb Graduate Research Fellowship, for RS. The funder was not involved in the study design, collection, analysis, interpretation of data, the writing of this article or the decision to submit it for publication.​​​​​

## Conflict of Interest

The authors declare that the research was conducted in the absence of any commercial or financial relationships that could be construed as a potential conflict of interest.
